# Features and outcomes of patients admitted to the ICU for chimeric antigen receptor T cell-related toxicity: a French multicentre cohort

**DOI:** 10.1186/s13613-024-01247-9

**Published:** 2024-01-31

**Authors:** Corentin Le Cacheux, Audrey Couturier, Clara Sortais, Roch Houot, Morgane Péré, Thomas Gastinne, Amélie Seguin, Jean Reignier, Jean-Baptiste Lascarrou, Jean-Marc Tadié, Quentin Quelven, Emmanuel Canet

**Affiliations:** 1 Service de Médecine Intensive Réanimation, Centre Hospitalier Universitaire Hôtel-Dieu, 30 Bd. Jean Monnet, 44093 Nantes Cedex 1, France; 2 Clinical Haematology Department, Rennes University Hospital, Rennes University, INSERM U1236, Rennes, France; 3 Haematology Department, Nantes University Hospital, Nantes University, Nantes, France; 4 Biostatistics Department, Nantes University Hospital, Nantes University, Nantes, France; 5 ICU, Nantes University, Nantes University Hospital,—Interactions—Performance Research Unit (MIP, UR 4334), Nantes, France; 6 ICU, Rennes University Hospital, Rennes University, Rennes, France

**Keywords:** Chimeric antigen receptor T Cell, Intensive care unit, Cytokine release syndrome, Immune effector cell-associated neurotoxicity syndrome, Haemophagocytic lymphohistiocytosis, Sepsis, Mortality

## Abstract

**Background:**

Chimeric antigen receptor T-cell (CAR-T) therapy is increasingly used in patients with refractory haematological malignancies but can induce severe adverse events. We aimed to describe the clinical features and outcomes of patients admitted to the intensive care unit (ICU) after CAR-T therapy.

**Methods:**

This retrospective observational cohort study included consecutive adults admitted to either of two French ICUs in 2018–2022 within 3 months after CAR-T therapy.

**Results:**

Among 238 patients given CAR-T therapy, 84 (35.3%) required ICU admission and were included in the study, a median of 5 [0–7] days after CAR-T infusion. Median SOFA and SAPSII scores were 3 [2–6] and 39 [30–48], respectively. Criteria for cytokine release syndrome were met in 80/84 (95.2%) patients, including 18/80 (22.5%) with grade 3–4 toxicity. Immune effector cell-associated neurotoxicity syndrome (ICANS) occurred in 46/84 (54.8%) patients, including 29/46 (63%) with grade 3–4 toxicity. Haemophagocytic lymphohistiocytosis was diagnosed in 15/84 (17.9%) patients. Tocilizumab was used in 73/84 (86.9%) patients, with a median of 2 [1–4] doses. Steroids were given to 55/84 (65.5%) patients, including 21/55 (38.2%) given high-dose pulse therapy. Overall, 23/84 (27.4%) patients had bacterial infections, 3/84 (3.6%) had fungal infections (1 invasive pulmonary aspergillosis and 2 *Mucorales*), and 2 (2.4%) had cytomegalovirus infection. Vasopressors were required in 23/84 (27.4%), invasive mechanical ventilation in 12/84 (14.3%), and dialysis in 4/84 (4.8%) patients. Four patients died in the ICU (including 2 after ICU readmission, i.e., overall mortality was 4.8% of patients). One year after CAR-T therapy, 41/84 (48.9%) patients were alive and in complete remission, 14/84 (16.7%) were alive and in relapse, and 29/84 (34.5%) had died. These outcomes were similar to those of patients never admitted to the ICU.

**Conclusion:**

ICU admission is common after CAR-T therapy and is usually performed to manage specific toxicities. Our experience is encouraging, with low ICU mortality despite a high rate of grade 3–4 toxicities, and half of patients being alive and in complete remission at one year.

**Supplementary Information:**

The online version contains supplementary material available at 10.1186/s13613-024-01247-9.

## Background

Chimeric antigen receptor T-cell (CAR-T) therapy is an innovative approach for managing refractory haematological malignancies. Autologous cytotoxic T lymphocytes are genetically modified to specifically recognise a tumour antigen, thereby causing tumour lysis [1, 2]. CAR-T therapy, first approved by the Food and Drug Administration in 2017 and by the European Medicines Agency in 2018, has provided extended survival and complete remission in patients with relapsed or refractory diffuse large B-cell lymphoma [3, 4] or acute lymphoblastic leukaemia (ALL) [5]. With newer indications such as multiple myeloma (MM) [6, 7], earlier treatment in lymphoma [8], and ongoing clinical trials in patients with solid tumours [9, 10], the number of patients given CAR-T therapy is predicted to increase steadily.

However, the inflammatory response generated by activated CAR-T cells can lead to potentially life-threatening complications, namely, cytokine release syndrome (CRS) and immune effector cell-associated neurotoxicity syndrome (ICANS). CRS is the most frequent, developing in up to 93% patients [3–5], and is defined as a febrile capillary leak syndrome, with hypotension, hypoxia, and organ failures in severe cases [11]. ICANS occurs in 20%–60% of patients, usually starting a few days after CRS onset [3–5, 12]. The manifestations encompass many neurological symptoms such as confusion, focal deficits, status epilepticus, and coma [13–15]. In addition, CAR-T recipients are severely immunocompromised and, therefore, at high risk for sepsis [16, 17].

Thus, several complications of CAR-T therapy may require intensive care unit (ICU) management. Overall, the outcomes of patients with haematological malignancies admitted to the ICU have improved substantially over the past two decades [18], but studies specifically addressing patients given CAR-T therapy are limited [19–21].

The objective of this retrospective observational study done in two ICUs was to assess the clinical features and outcomes of patients requiring ICU admission after CAR-T therapy. We hypothesised that our findings would support the use of ICU resources for such patients.

## Methods

The study was approved by the ethics committee of the French Intensive Care Society (CE SRLF 22-044) on July 7, 2022. In accordance with French law on retrospective studies of anonymized healthcare data, informed consent was not required. This report complies with STROBE guidelines [22] (Additional file 1: Appendix 1).

### Study design and population

We retrospectively identified consecutive adults (≥ 18 years) who were admitted to either of two university-hospital ICUs, in Nantes and Rennes (France), respectively, between 1 August 2018 and 31 May 2022 and within 90 days after receiving CAR-T therapy. For patients admitted more than once during the study period, data were collected for each ICU stay. The total number of ICU stays was used to describe the full spectrum of infections in the supplementary appendix and to describe and investigate the subset of patients who were readmitted to the ICU during the study period. We used the haematology department database to identify patients given CAR-T therapy during the study period. The list of patients thus obtained was cross-referenced with that of patients in the ICU database in each hospital to identify patients admitted to the ICU within 90 days after CAR-T therapy. One author (CLC) reviewed the medical file of each patient thus identified to check that the inclusion criteria were met. Of note, 27 patients of the present study (7 in Nantes and 20 in Rennes) were included in the CARTTAS study [19]. During the study period, both ICUs applied an unrestricted admission policy for patients given CAR-T therapy.

### Data collection

For each patient, the data reported in Tables 1, 2, and 3 were extracted from the ICU records and entered by the local investigator at each ICU into a standardised electronic case-report form (Additional file 1: Appendix 2).

Diagnoses of CRS and ICANS were based on medical records, with each case reassessed by investigators in each centre and graded according to the American Society for Transplantation and Cellular Therapy (ASTCT) consensus grading system [23] (Additional file 1: Appendix 3). The CAR-T therapy-associated toxicity (CARTOX) score [24] and Immune effector Cell-associated Encephalopathy (ICE) score [23] (Additional file 1: Appendix 4) were recorded when available in the medical charts. Infections were categorised as either microbiologically documented (identification of a pathogen on samples taken between symptoms onset and ICU discharge) or clinically documented (identification of a clinical site of infection not accessible to sampling or sampled with negative microbiological results). Sepsis was diagnosed according to the Sepsis-3 criteria [25]. Haemophagocytic lymphohistiocytosis (HLH) was reported in the event of a diagnosis made by the attending physicians and recorded in the medical record as such, with a specific review of blood parameters and diagnostic investigations in each case. Of note, diagnoses of CRS, sepsis and HLH were not considered as mutually exclusive. Neutropenia was defined as a white-blood-cell count below 500/mm^3^ and thrombocytopenia as a platelet count below 100 000/mm^3^. Disease severity was assessed using the Simplified Acute Physiology Score (SAPS II) [26] and the Sequential Organ Failure Assessment (SOFA) score [27] on day 1 after ICU admission. CRS and ICANS were treated according to the guidelines of the French Society of Bone Marrow Transplantation and Cellular Therapy [28–31]. One-year outcomes after CAR-T infusion (vital status, and haematological status defined as complete remission, partial remission, or disease progression) were recorded for all patients given CAR-T therapy in both hospitals, to allow comparison between patients with vs. without ICU admission.

### Objectives

The primary objective of the study was to describe the clinical features, treatments, and outcomes of patients who became critically ill after receiving CAR-T therapy. The secondary objective was to report the one-year outcomes (overall survival and progression-free survival) of patients who were and were not admitted to the ICU after CAR-T therapy.

### Statistical analysis

Continuous variables were described as median [interquartile range] and compared between groups using the non-parametric Mann–Whitney test. Categorical variables were described as frequency (percentages) and compared between groups using Fisher’s exact test. Mortality and haematological status were assessed by survival analysis. Kaplan–Meier graphs were plotted to express the probability of death or disease progression from intensive care unit admission to one-year follow-up, and comparisons were done using the log-rank test. Missing data were recorded but not imputed. All tests were two-sided, and *p* values lower than 5% were considered to indicate significant associations. Statistical tests were conducted using the R statistics programme, version 3.5.0 (R Foundation for Statistical Computing, Vienna, Austria; www.R-project.org/).

## Results

### Study population

Among 238 patients given CAR-T therapy during the study period, 84 (35.3%) required ICU admission and were included in the analysis, with a total of 97 ICU stays (Additional file 1: Appendix 5). Table 1 reports the main characteristics of these 84 patients. Median time from CAR-T infusion to ICU admission was 5 days [0–7]. The main reasons for ICU admission (97 stays) were neurological failure (36.1%), haemodynamic instability (29.9%), and close monitoring (34%). Median SOFA and SAPSII scores were 3 [2–6] and 39 [30–48], respectively. Most patients had few non-malignant comorbidities and were in good general health, with 92.7% having a performance status of 0–2. All patients received lymphodepleting chemotherapy with cyclophosphamide and fludarabine before CAR-T therapy. At ICU admission, 70 (83.3%) patients had neutropenia and 56 (66.7%) had thrombocytopenia.

**Table 1:** Baseline characteristics of the 84 study participants

Baseline patient characteristics	N (%) or Median [IQR]
**Demographics**	
Age, years	64 [50–69.8]
Male	47 (56)
Body mass index	24 [21.6–27] ^a^
**General health**	
Charlson Comorbidity Index	4 [2–5]
ECOG Performance Status	1 [0–1.25]
*0*	*21 (25)*
*1–2*	*56 (66.7)*
*3–4*	*7 (8.3)*
Clinical Frailty Scale score	2 [2–4]
**Haematological malignancy**	
*Diffuse large B-cell lymphoma*	*54 (64.3)*
*Other lymphoma*	*16 [19]*
*B-cell acute lymphoblastic leukaemia*	*8 (9.5)*
*Multiple myeloma*	*6 (7.1)*
Time from hematological diagnosis to CAR-T therapy, months	17.6 [8.8–37.5]
Number of chemotherapy lines before CAR-T therapy	2.5 [2, 3]
Autologous haematopoietic stem-cell transplantation	18 (21.4)
Allogeneic haematopoietic stem-cell transplantation	8 (9.5)
**CAR-T product**	
*Axicabtagene ciloleucel*	*62 (73.8)*
*Tisagenlecleucel*	*10 (11.9)*
*Brexucabtagen autoleucel*	*6 (7.1)*
*Idecabtagene vicleucel*	*6 (7.1)*

^a^ Data missing in 2 patients

### Characteristics of CAR-T toxicity and ICU management

**Fig. 1:**
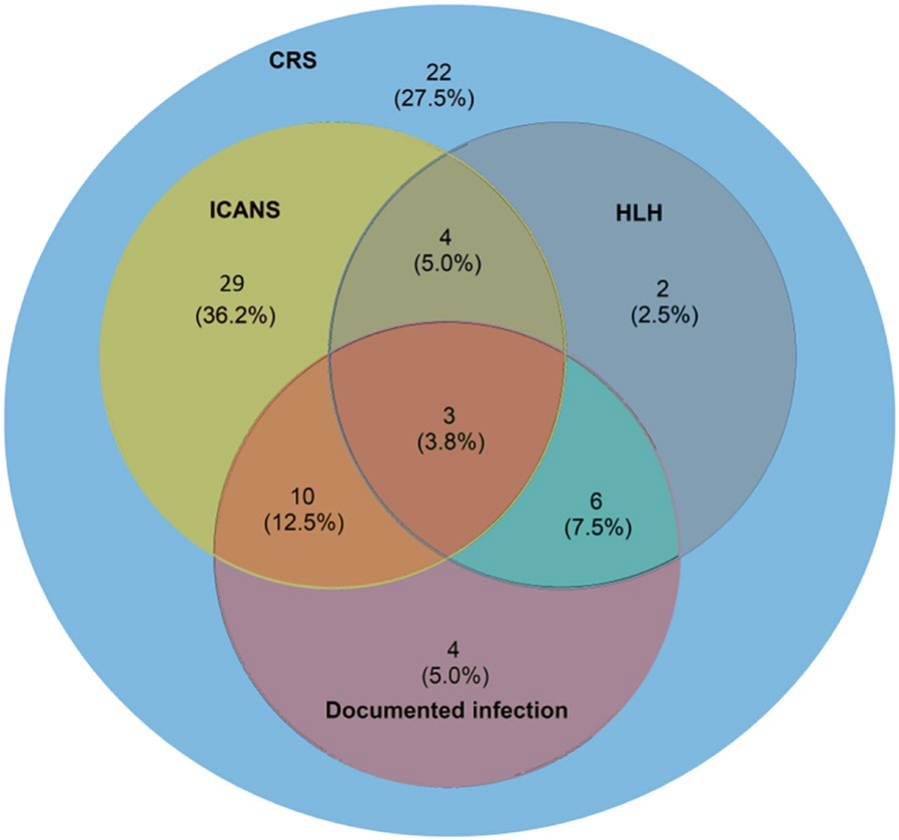
Spectrum of CAR-T therapy complications diagnosed in the ICU. *CAR-T* Chimeric antigen receptor-T cells; *ICU* intensive care unit; *CRS* cytokine release syndrome; *ICANS* Immune effector cell-associated neurotoxicity syndrome; *HLH* haemophagocytic lymphohistiocytosis

Table 2 and Fig. 1 report the diagnoses and ICU management. Time from CAR-T infusion to onset of any symptom attributable to CRS or ICANS was 2 [1–5] days after CAR-T infusion and time from symptom onset to ICU admission was 3 [1–5] days.

**Table 2:** Occurrence and characteristics of cytokine release syndrome (CRS) and immune effector cell-associated neurotoxicity syndrome (ICANS) in the 84 patients

	**N (%) or** **Median [IQR]**
**Patients with CRS**	**80 (95.2)**
CRS grade (n = 80) ^a^	
*1*	*33/80 (41.3)*
*2*	*29/80 (36.3)*
*3*	*15/80 (18.8)*
*4*	*3/80 (3.8)*
Fever duration (days)	5 [3–7]
Time from fever to hypotension (days)	2 [1–3.75]
Time from fever onset to peak CRS severity (days)	5 [2–7]
CRS-associated complications	
*HLH without DIC*	*8/*80 (10)
*HLH and DIC*	*7/80 (8.8)*
*DIC without HLH*	*1/80 (1.3)*
**Patients with ICANS**	**46 (54.8)**
ICANS grade (n = 46) ^a^	
*1*	*9/46 (19.6)*
*2*	*8/46 (17.4)*
*3*	*15/46 (32.6)*
*4*	*14/46 (30.4)*
Time to neurological symptoms resolution (days)	5 [3–8]
Time from CRS to ICANS (days) ^b^	3 [2–4]
Time from neurological symptoms onset to peak ICANS severity (days)	1 [0–2]
**Investigations for ICANS**	
Cerebral computed tomography	37/46 (80.4)
*Basal ganglia hypodensities*	*2/37 (5.4)*
Cerebral magnetic resonance imaging	29/46 (63)
*Abnormal findings* ^c^	*11/29 (37.9)*
Lumbar puncture	36/49 (78.3)
*Abnormal findings*	*25/36 (69.4)*
*Protein elevation ≥ 0.5 g/L*	*25/36 (69.4)*
*Pleocytosis ≥ 10/mm*^3^	*9/36 (25)*
Electroencephalography	39/46 (84.8)
*Abnormal findings*	*39/39 (100)*
*Non-specific encephalopathy*	*33/39 (84.6)*
*Seizures*	*6/39 (15.4)*
*Status epilepticus*	*3/39 (7.7)*

*HLH* Haemophagocytic lymphohistiocytosis, *DIC* disseminated intravascular coagulation

^a^ The maximum grade for each patient is reported

^b^ All patients with ICANS had CRS

^c^ Basal ganglia and/or white matter FLAIR hypersignals with inconsistent contrast enhancement

Of the 84 patients, 80 (95.2%) met CRS criteria, including 18 (22.5%) who had CRS grade 3 or 4. In these 18 patients, time from CAR-T infusion to symptom onset was significantly shorter than in the 62 patients with grade 1 or 2 CRS (1 [0–3] vs. 3 [1–5] days, *p* = 0.023).

ICANS was diagnosed in 46/84 (54.8%) patients. All 46 had CRS and 29 (63%) had grade 3–4 ICANS. Of the 46 patients, 29 (63%) experienced worsening of their neurological condition after ICU admission. The most common neurological symptoms were confusion (95.7%), decreased level of consciousness (65.9%), aphasia (47.8%), and focal signs (47.8%). All ICANS patients in whom cerebral imaging investigations revealed abnormalities had grade 3–4 ICANS. No patient had clinical or radiological signs of intracranial hypertension, whereas 8/46 (17.4%) had seizures, including 3 with status epilepticus. ICANS lasted more than 14 days in 8 patients.

Overall, 42/84 (50%) patients had grade 3–4 CRS and/or ICANS and 5 (6%) had both grade 3–4 CRS and grade 3–4 ICANS.

Of the 84 patients, 23 (27.4%) had microbiologically documented infections, which were chiefly bacterial infections from pulmonary and/or gastrointestinal sources (Additional file 1: Appendix 6). Urgent, empirical broad-spectrum antibiotics were used during 86/97 (88.7%) ICU stays and in all 80 patients with CRS. Cytomegalovirus disease was diagnosed in 2 patients and invasive fungal infections in 3 patients (1 with aspergillosis and 2 with *Mucorales* infection). Neutropenia duration was 7 [5-25] days, and 25/84 (29.8%) patients experienced relapsing cytopenia. Haemophagocytic lymphohistiocytosis (HLH) was diagnosed in 15/84 (17.9%) patients (Additional file 1: Appendix 7). No patient had tumour lysis syndrome. Fifteen (17.9%) patients developed acute kidney injury in the ICU (KDIGO stage 1: n = 6, stage 2: n = 3, stage 3: n = 6).

Tocilizumab was administered to 73/84 (86.9%) patients and corticosteroids to 55/84 (65.5%) patients, including 21/55 (38.2%) given high-dose pulse therapy. Second-line treatment was given to 7/84 (8.3%) patients and consisted of siltuximab (n = 4), anakinra (n = 2), or both (n = 1) (details are provided in Additional file 1: Appendix 8). Life-supporting interventions (Table 3) were implemented in 27/84 (32.1%) patients, the most common being vasopressor administration (23/84, 27.4%), followed by invasive mechanical ventilation (12/84, 14.3%) and renal replacement therapy (4/84, 4.8%).

**Table 3:** Treatments used in the intensive care unit (ICU) in the 84 patients

	**N (%) or Median [IQR]**
**Tocilizumab**	**73 (86.9)**
*Time from initiation to ICU admission (days)*	*1 (− 1.5–3)*
*Initiated before ICU admission*	*53/73 (72.6)*
*Number of injections*	*2 [1–4]*
**Corticosteroids** ^a^	**55 (65.5)**
*Time from initiation to ICU admission (days)*	*0 (0–1)*
*Initiated before ICU admission*	*41/55 (74.5)*
*Cumulative dose in the ICU (mg prednisone-equivalent)*	*2400 [1120–4750]*
**Tocilizumab and Corticosteroids**	**52 (61.9)**
Granulocyte growth factors	59 (70.2)
Anti-epileptic drug prophylaxis	81 (96.4)
Intravenous fluids, mL, median within 3 days after admission	1000 [875-1000} ^b^
Vasopressors	23 (27.4)
*Duration (days)*	*2 [1–4]*
*Maximum dose (µg/kg/min)*	*0.3 [0.2–0.5]*
Invasive mechanical ventilation	12 (14.3)
*Duration (days)*	*4 [3–7]*
Renal replacement therapy	4 (4.8)
*Duration (days)*	*3 [1.75–8.5]*

*ICU* Intensive care unit, *ICANS* Immune effector cell-associated neurotoxicity syndrome, *CRS* Cytokine release syndrome

^a^Indication: ICANS (40/55); Refractory CRS (15/55)

^b^Data missing in 2 patients

### Outcomes

ICU and hospital lengths of stay were 4 (2.0–5.25) days and 24 (20.0–33.75) days, respectively. ICU readmission was required for 12 (14.3%) patients (Additional file 1: Appendix 9), mostly due to sepsis or ICANS relapse, of whom 2 (16.7%) died during the second ICU stay. Of the 84 patients, 4 died in the ICU (including 2 who died during readmission) (Additional file 1: Appendix 10), yielding an overall mortality rate of 4.8% of patients. In-hospital mortality was 11.9% (n = 10/84).

**Fig. 2:**
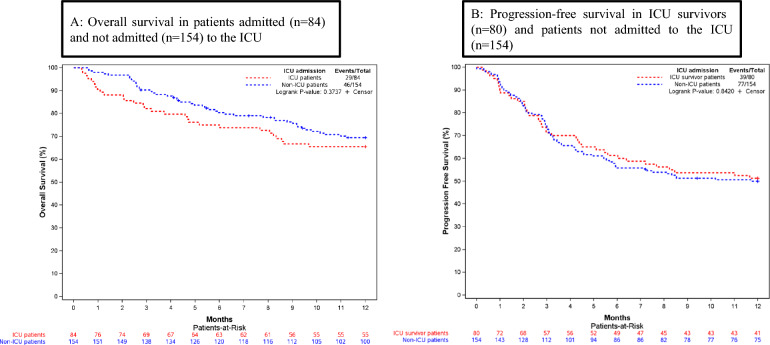
Analysis of one-year Overall Survival (**A**) and Progression-Free Survival (**B**) in patients admitted and not admitted to the ICU. *ICU* Intensive care unit. 2A: Shown are Kaplan–Meier estimates of overall survival among the patients admitted and not admitted to the ICU. 2B: Shown are Kaplan–Meier estimates of progression-free survival among the ICU survivors and the patients not admitted to the ICU

At one-year follow-up, outcomes in the 80 ICU survivors were similar to those in the 154 patients without ICU admission (*p* = 0.84) (Fig. 2). Of the 80 surviving patients, 41 (51.3%) were in complete remission, 14 (17.5%) had disease progression, and 25 (31.3%) had died.

## Discussion

### Key findings

Among CAR-T recipients, about a third required ICU admission for haemodynamic instability or neurological failure a few days after the infusion. CRS and ICANS were the main reasons for ICU admission. Sepsis was diagnosed in a quarter of the patients. Despite a frequent need for life-supporting interventions, most patients recovered within a week and ICU mortality was less than 5%. Moreover, ICU survivors had similar haematological outcomes to those in CAR-T recipients not admitted to the ICU.

### Comparison to previous studies

Despite the increasing use of CAR-T therapy for a variety of haematological malignancies, knowledge on the ICU management and outcomes of CAR-T recipients comes only from a small number of expert centres [19–21]. All these centres reported a high ICU admission rate (27% to 35%) following CAR-T therapy. The proportion was 35% in our study. These data highlight the key role for critical care in CAR-T recipients, mainly early after the infusion. In two earlier studies [19, 21], ICU admission for hypotension was more common than in our cohort (70% and 48%, respectively, vs. 30%). However, the vasopressor requirements were similar, suggesting differences in ICU admission policies rather than in illness severity.

CRS and ICANS are common after CAR-T administration and constitute the main reasons for ICU admission. Our finding that 95% of patients met criteria for CRS and 55% those for ICANS is in keeping with previous studies [19–21]. All our patients with ICANS also had CRS, in accordance with current pathophysiological knowledge [32, 33], although in the international CARTTAS cohort, 7/238 patients had ICANS without CRS [19]. The treatment of CRS relies on the IL-6 receptor antagonist tocilizumab and on corticosteroids as a second-line treatment, whereas corticosteroids are the recommended first-line treatment for ICANS [30, 31]. All ICU studies found similar rates of tocilizumab and corticosteroids use, but with major differences across centres regarding the drugs chosen, doses, timing of administration and discontinuation, and additional treatments [19–21]. Although guidelines have been issued, current recommendations rely on expert opinion and observational data, as no randomised controlled trials are available. Concern has been expressed regarding potential deleterious effects of high-dose corticosteroids on the efficacy of CAR-T therapy [20, 34], as the overwhelming majority of deaths after CAR-T infusion are due to disease progression or relapse [19, 34, 35]. Further studies are therefore needed to define the optimal management of CRS and ICANS.

HLH developed in 17.9% of our patients, compared to 3.8%–5% in previous reports [19, 20]. This discrepancy may be related to differences in the underlying malignancies, type of CAR-T used, and diagnostic criteria for HLH. This syndrome is challenging to distinguish from CRS and sepsis in clinical practice [30]. A recent study emphasised the need for further research to better recognize, define, and treat HLH in CAR-T recipients [36].

As ICU admission usually occurs early after CAR-T therapy, during the neutropenic phase, sepsis is a major concern and the main differential diagnosis of CRS in daily practice. The CARTTAS study found bacterial infection to be independently associated with a twofold higher mortality rate [19]. Microbiologically documented sepsis has been reported to occur in 16% to 30% of patients, in keeping with the 27% proportion in our cohort [19–21]. Three of our patients had fungal infections. The optimal prophylactic or pre-emptive strategy for infection after CAR-T therapy has yet to be determined but as of now, urgent broad-spectrum antibiotics in all febrile patients with neutropenia remain essential [37, 38].

Finally, although severe CRS and ICANS may require life-supporting interventions, recovery usually occurs within a week. ICU admission after CAR-T therapy was consistently associated with greater than 90% survival at ICU discharge [19–21]. Our experience was similar, with an overall mortality rate of 4.8%. Nonetheless, the subset of patients who required ICU readmission had a higher mortality. Importantly, ICU survivors had similar one-year outcomes to those of patients not admitted to the ICU.

### Study implications

Our findings of excellent in-ICU and one-year outcomes, despite a substantial rate of high-grade toxicities following CAR-T therapy, support early unrestricted ICU admission without undue concern about a possible negative impact of critical care on CAR-T efficacy and the haematological prognosis. Moreover, our results imply that because sepsis is common and indistinguishable from CRS in clinical practice, sepsis can never be ruled out during the neutropenic phase and should be thoroughly investigated, and broad-spectrum antibiotics administered without delay. Finally, despite rapid advances in pathophysiological understanding, the main immunomodulatory treatments and specific therapies used to control CAR-T toxicities remain largely empirical. The prospective collection of data in nationwide registries would increase the amount of available data, thereby providing a strong basis for further research.

### Strengths and limitations

Our study has several limitations. First, the difficulty of distinguishing CRS from sepsis carries a risk of adjudication bias, particularly given the retrospective design. Similarly, confounding factors such as uraemia, high fever, and use of beta-lactams or neurotropic agents may lead to ICANS-like symptoms. However, in the absence of a reference-standard diagnostic strategy, we strictly applied ASTCT grading recommendations for CRS and ICANS and reported microbiologically documented infections, refraining from classifying patients based on subjective criteria. Second, we included patients over a nearly 4-year period, during which both critical-care and haematology teams gained experience in managing CAR-T therapy recipients. The criteria for transferring these patients to the ICU may therefore have changed over the recruitment period. Our results may not apply to other ICUs with different admission policies. However, our study adds relevant and comprehensive data from two experienced ICUs that were among the first to treat CAR-T therapy recipients in France. Third, the study design prevented us from evaluating how the specific treatments used in the ICU may have affected patient outcomes. However, current recommendations rely chiefly on low-level observational evidence. Fourth, more than 70% of patients were treated with axicabtagen ciloleucel, and our findings may not apply to patients treated with other CAR products. Finally, the very low mortality precluded a multivariable analysis designed to identify independent predictors of death.

## Conclusion

In conclusion, our study confirms that intensive care is an integral part of the management of patients given CAR-T therapy. Both specific toxicities (CRS, ICANS, and HLH) and sepsis may require intensive care. The short-term outcomes are excellent, and critical care is not associated with worse one-year haematological outcomes. Studies are needed to investigate the interplay between CAR-T efficacy, toxicity, and the impact of immunomodulating treatments. Moreover, as the current standard of care remains largely empirical, interventional studies are now needed to guide clinical practice.

### Supplementary Information

**Additional file 1:** **Appendix 1:** STROBE Statement. **Appendix 2:** Supplementary methods.** Appendix 3:** American Society for Transplantation and Cellular Therapy (ASTCT) grading for CRS and ICANS. **Appendix 4:** Encephalopathy Assessment Tools for Grading of ICANS: CARTOX-10 and ICE scores. **Appendix 5**: Study flowchart. **Appendix 6:** Infectious complications during the 97 ICU stays in 84 patients. **Appendix 7: **Additional data on patients with haemophagocytic lymphohistiocytosis (HLH) (n=15).** Appendix 8:** Use of second-line immunosuppressors. **Appendix 9:** Additional data on patients with repeated admissions.** Appendix 10:** Causes of death in CAR-T recipients

## Data Availability

The datasets used and/or analysed during the current study are available from the corresponding author on reasonable request.

## Abbreviations

CAR-T: Chimeric antigen receptor T-cell
CRS: Cytokine release syndrome
HLH: Haemophagocytic lymphohistiocytosis
ICANS: Immune effector cell-associated neurotoxicity syndrome
ICU: Intensive care unit
