# Supplementary material for: Features and outcomes of patients admitted to the ICU for chimeric antigen receptor T cell-related toxicity: a French multicentre cohort
Source: Ann Intensive Care. 2024 Jan 31;14:20. doi: 10.1186/s13613-024-01247-9 (PMC10828176; doi:10.1186/s13613-024-01247-9)
Supplement: Supplementary file 1 — Additional file 1: Appendix 1: STROBE Statement. Appendix 2: Supplementary methods. Appendix 3: American Society for Transplantation and Cellular Therapy (ASTCT) grading for CRS and ICANS. Appendix 4: Encephalopathy Assessment Tools for Grading of ICANS: CARTOX-10 and ICE scores. Appendix 5: Study flowchart. Appendix 6: Infectious complications during the 97 ICU stays in 84 patients. Appendix 7: Additional data on patients with haemophagocytic lymphohistiocytosis (HLH) (n=15). Appendix 8: Use of second-line immunosuppressors. Appendix 9: Additional data on patients with repeated admissions. Appendix 10: Causes of death in CAR-T recipients [file 13613_2024_1247_MOESM1_ESM.docx]

**Additional file 1.**

**Appendix 1: STROBE Statement—checklist of items that should be included in reports of observational studies**

|  | Item No. | Recommendation | Page  No. |
| --- | --- | --- | --- |
| Title and abstract | 1 | (*a*) Indicate the study’s design with a commonly used term in the title or the abstract | 1 |
|  |  | (*b*) Provide in the abstract an informative and balanced summary of what was done and what was found | 2 |
| Introduction | | | |
| Background/rationale | 2 | Explain the scientific background and rationale for the investigation being reported | 3 and 4 |
| Objectives | 3 | State specific objectives, including any prespecified hypotheses | 4 |
| Methods | | | |
| Study design | 4 | Present key elements of study design early in the paper | 5 |
| Setting | 5 | Describe the setting, locations, and relevant dates, including periods of recruitment, exposure, follow-up, and data collection | 5 |
| Participants | 6 | (*a*) *Cohort study*—Give the eligibility criteria, and the sources and methods of selection of participants. Describe methods of follow-up | 5 |
|  |  | (*b*) *Cohort study*—For matched studies, give matching criteria and number of exposed and unexposed | NA |
| Variables | 7 | Clearly define all outcomes, exposures, predictors, potential confounders, and effect modifiers. Give diagnostic criteria, if applicable | 6 |
| Data sources/ measurement | 8* | For each variable of interest, give sources of data and details of methods of assessment (measurement). Describe comparability of assessment methods if there is more than one group | 5 and 6 |
| Bias | 9 | Describe any efforts to address potential sources of bias | 5 |
| Study size | 10 | Explain how the study size was arrived at | NA |
| Quantitative variables | 11 | Explain how quantitative variables were handled in the analyses. If applicable, describe which groupings were chosen and why | 6 and 7 |
| Statistical methods | 12 | (*a*) Describe all statistical methods, including those used to control for confounding | 7 |
|  |  | (*b*) Describe any methods used to examine subgroups and interactions | NA |
|  |  | (*c*) Explain how missing data were addressed | 7 |
|  |  | (*d*) *Cohort study*—If applicable, explain how loss to follow-up was addressed | 7 |
|  |  | (*e*) Describe any sensitivity analyses | NA |
| Participants | 13* | (a) Report numbers of individuals at each stage of study—eg numbers potentially eligible, examined for eligibility, confirmed eligible, included in the study, completing follow-up, and analysed | e-Appendix 5 |
|  |  | (b) Give reasons for non-participation at each stage | NA |
|  |  | (c) Consider use of a flow diagram | e-Appendix 5 |
| Descriptive data | 14* | (a) Give characteristics of study participants (eg demographic, clinical, social) and information on exposures and potential confounders | Table 1 |
|  |  | (b) Indicate number of participants with missing data for each variable of interest | Table 1, Table 3 |
|  |  | (c) *Cohort study*—Summarise follow-up time (eg, average and total amount) | 9 |
| Outcome data | 15* | *Cohort study*—Report numbers of outcome events or summary measures over time | Table 2, Table 3, Figure 2 |
| Main results | 16 | (*a*) Give unadjusted estimates and, if applicable, confounder-adjusted estimates and their precision (eg, 95% confidence interval). Make clear which confounders were adjusted for and why they were included | NA |
|  |  | (*b*) Report category boundaries when continuous variables were categorized | NA |
|  |  | (*c*) If relevant, consider translating estimates of relative risk into absolute risk for a meaningful time period | NA |
| Other analyses | 17 | Report other analyses done—eg analyses of subgroups and interactions, and sensitivity analyses | NA |
| Key results | 18 | Summarise key results with reference to study objectives | 10 |
| Limitations | 19 | Discuss limitations of the study, taking into account sources of potential bias or imprecision. Discuss both direction and magnitude of any potential bias | 12 and 13 |
| Interpretation | 20 | Give a cautious overall interpretation of results considering objectives, limitations, multiplicity of analyses, results from similar studies, and other relevant evidence | 10 to 13 |
| Generalisability | 21 | Discuss the generalisability (external validity) of the study results | 13 |
| Other information |  |  |  |
| Funding | 22 | Give the source of funding and the role of the funders for the present study and, if applicable, for the original study on which the present article is based | 14 |

**Appendix 2: Supplementary methods**

**Patient recruitment:**

At the time of this study and based on French hospital administration, only accredited, university hospital-affiliated haematology departments were performing CAR-T therapy. Similarly, all CAR-T recipients requiring ICU admission were referred to a single specialized ICU in each center, due to inter-departmental agreement and the need for expertise. Therefore, all haematology patients requiring CAR-T therapy in the area were treated exclusively in the haematology department of Nantes or Rennes university hospitals, respectively. Moreover, all CAR-T recipients requiring ICU admission in Nantes or Rennes university hospitals were exclusively referred to the participating ICU in each center.

**Data collection:**

The following data were collected for our study:

- age and sex; body mass index; Charlson Comorbidity Index; presence of hypertension, diabetes, alcohol abuse, tobacco use; and previous self-sufficiency assessed by the Clinical Frailty Scale and ECOG Performance Status

- haematological and medical history: nature and date of diagnosis of the haematological malignancy for which CAR-T was given; number and nature of prior chemotherapy lines; history of autogenous or allogeneic hematopoietic stem-cell transplantation; date of lymphodepletion, nature of the CAR-T product; date of the CAR-T infusion; dates of admission and discharge from the hospital and intensive care unit; dates of onset and resolution of aplasia; and time from CAR-T infusion to onset of toxicity symptoms

- ICU admission: times from CAR-T infusion and from CAR-T toxicity symptom onset to admission; reason for admission; clinical parameters on admission (heart rate, blood pressure, respiratory rate, oxygen saturation, body temperature, Glasgow Coma Scale score, and need for and nature of oxygen therapy); severity scores (SAPS II and SOFA score) 24 hours after ICU admission; and presence and nature of central venous lines

- CRS characteristics: dates of fever and hypotension onset and resolution; initial and maximum in-ICU grade with the dates; laboratory variables at peak severity (blood cell counts, lactate, liver enzymes, bilirubinaemia, coagulation parameters, fibrinogen, D-dimers, C-reactive protein, lactic dehydrogenase, serum uric acid, ferritin, triglycerides); and thoracic ultrasound evaluation and findings if applicable

- ICANS characteristics: times of onset and resolution; initial and peak in-ICU grade with the dates; nature of the symptoms; consecutive ICE/CARTOX scores; specific investigations (lumbar puncture, neuroimaging, electroencephalography) with the modalities and findings; whether antiepileptic treatment was given; and specific records for convulsions, status epilepticus, and intracranial hypertension

- sepsis diagnosis and management: presence of a microbiologically documented infection (identification of a pathogen) or clinically documented infection (identification of a clinical site of infection not accessible to sampling or sampled with negative microbiological results) before and/or during ICU admission (with separate records for each); source of infection; pathogen identification; nature of samples; presence and nature of curative and prophylactic anti-infectious treatment on the ward and at ICU admission (separate records for each); efficacy of empirical treatment at the time microbiological documentation was obtained

- life-supporting interventions: fluid resuscitation volume during the first 3 days in the ICU, presence and nature of vasopressors (with the dates of initiation and weaning and the maximum dose); presence of acute kidney injury (with the KDIGO grade and use of renal replacement therapy with the dates of initiation and weaning); type of respiratory assistance required, notably use of invasive mechanical ventilation with the dates of initiation and weaning

- specific treatments: use before ICU admission or in the ICU (separate records) of corticosteroid therapy with the date of initiation, type, cumulative dose in the ICU; use before ICU admission or in the ICU (separate records) of tocilizumab with the date of initiation and cumulative dose; use in the ICU of other immunosuppressants (with the nature and date of initiation); use of blood transfusions and/or hematopoietic growth agents

- outcome data: infectious complications (open record); haematological complications (both open record and specific records for disseminated intravascular coagulation, haemophagocytic lymphohistiocytosis [with a specific review of blood parameters and diagnostic investigations], and tumour lysis syndrome); other complications (open record); whether treatment limitation was decided and implemented, with the dates; vital status at ICU discharge; whether the patient was readmitted to the ICU, with the date; vital and haematological status at one year with the date of death or progression/relapse (recorded by the haematologists as part of the standard follow-up), and cause of death if relevant.

**Medical management of CRS and ICANS:**

CRS and ICANS were treated throughout the study according to the current guidelines of the French Society of Bone Marrow Transplantation and Cellular Therapy (1-4). In all versions of these guidelines, tocilizumab was used as first-line treatment for isolated CRS (persistent grade 1, or grade 2 to 4), with corticosteroids as second-line treatment, allowing dose escalation up to the use of high-dose pulsed therapy in refractory and life-threatening cases. For ICANS (with or without CRS), corticosteroids were used as first-line therapy, with high-dose pulsed therapy in refractory or life-threatening cases. Other immunomodulators (such as siltuximab and anakinra) were used as rescue therapy in CRS and ICANS. No immunomodulators were used prophylactically.

Granulocyte growth factors were used in stabilized patients with clinical improvement, for whom discharge from intensive care was being considered but who had persistent neutropenia, in the absence of uncontrolled infection.

Anti-epileptic drug prophylaxis (levetiracetam) was used in a protocolized manner in both haematology departments in patients with worsening clinical condition attributed to CRS or ICANS, for whom transfer to the ICU was considered. Such treatment was pursued in the ICU in the absence of suspected drug-induced toxicity.

1. Yakoub-Agha I, Moreau AS, Ahmad I, Borel C, Hadhoum N, Masouridi-Levrat S, et al. Prise en charge pratique du syndrome de relargage des cytokines (CRS) post-CAR-T cells chez l’adulte et l’enfant : recommandation de la Société francophone de greffe de moelle et de thérapie cellulaire (SFGM-TC). Bull Cancer (Paris). 2019;106:S102–9. [In French]

2. Cornillon J, Hadhoum N, Roth-Guepin G, Quessar A, Platon L, Ouachée-Chardin M, et al. Prise en charge pratique d’une encéphalopathie liée au traitement par cellules CAR-T chez l’adulte et l’enfant : recommandations de la Société francophone de greffe de moelle et de thérapie cellulaire (SFGM-TC). Bull Cancer (Paris). 2020;107:S12–7. [In French]

3. Tudesq JJ, Yakoub-Agha M, Bay JO, Courbon C, Paul F, Picard M, et al. [Management of cytokine release syndrome and macrophage activation syndrome following CAR-T cell therapy: Guidelines from the SFGM-TC]. Bull Cancer (Paris). 2023;110:S116–22. [In French]

4. Picard M, Sterin A, Bay JO, Courbon C, Moreau AS, Paul F, et al. [Management of neurotoxicity following CAR-T cell therapy: Recommendations of the SFGM-TC]. Bull Cancer (Paris). 2023;110:S123–31. [In French]

**Appendix 3: American Society for Transplantation and Cellular Therapy (ASTCT) grading for CRS and ICANS**

| **CRS grading** | **Grade 1** | **Grade 2** | **Grade 3** | **Grade 4** |
| --- | --- | --- | --- | --- |
| **Fever** | >38° C | >38° C | >38° C | >38° C |
| With | | | | |
| **Hypotension** | None | Not requiring vasopressors Requiring a vasopressor with or without vasopressin Requiring multiple vasopressors (excluding vasopressin) | Requiring a vasopressor with or without vasopressin | Requiring multiple vasopressors (excluding vasopressin) |
| And/or | | | | |
| **Hypoxia** | None | Requiring low-flow nasal cannula or blow-by mask | Requiring high-flow nasal cannula, facemask, nonrebreather mask, or Venturi mask | Requiring positive pressure (e.g., CPAP, BiPAP, intubation and mechanical ventilation) |

| **ICANS grading** | **Grade 1** | **Grade 2** | **Grade 3** | **Grade 4** |
| --- | --- | --- | --- | --- |
| **ICE score** | 7-9 | 3-6 | 0-2 | 0 (patient is unarousable and unable to perform ICE score) |
| **Level of consciousness** | Awakens spontaneously | Awakens to voice | Awakens only to tactile stimulus | Patient is unarousable or requires vigorous or repetitive tactile stimuli to arouse.  Stupor or coma |
| **Seizure** | NA | NA | Any clinical seizure focal or generalized that resolves rapidly or nonconvulsive seizures on EEG that resolve with intervention | Life-threatening prolonged seizure (>5 min); or Repetitive clinical or electrical seizures without return to baseline in between |
| **Motor findings** | NA | NA | NA | Deep focal motor weakness such as hemiparesis or paraparesis |
| **Elevated intracranial pressure/ cerebral oedema** | NA | NA | Focal/local oedema on neuroimaging | Diffuse cerebral oedema on neuroimaging; decerebrate or decorticate posturing; or cranial nerve VI palsy; or papilledema; or Cushing's triad |

CRS: cytokine release syndrome; ICANS: immune effector cell-associated neurotoxicity syndrome; NA: not applicable; ICE: immune effector cell encephalopathy; CPAP: continuous positive airway pressure; BiPAP: bilevel positive airway pressure; EEG: electroencephalogram

**Appendix 4: Encephalopathy Assessment Tools for Grading of ICANS: CARTOX-10 and ICE scores**

CARTOX-10

- Orientation: orientation to year, month, city, hospital, president/prime minister of country of residence: 5 points

- Naming: ability to name 3 objects (e.g., point to clock, pen, button): 3 points

- Writing: ability to write a standard sentence (e.g., “My tailor is rich”): 1 point

- Attention: ability to count backwards from 100 by 10: 1 point

ICE

- Orientation: orientation to year, month, city, hospital: 4 points

- Naming: ability to name 3 objects (e.g., point to clock, pen, button): 3 points

- Following commands: ability to follow simple commands (e.g., “Show me 2 fingers” or “Close your eyes and stick out your tongue”): 1 point

- Writing: ability to write a standard sentence (e.g., “My tailor is rich”): 1 point

- Attention: ability to count backwards from 100 by 10: 1 point

**Appendix 5: Study flowchart**


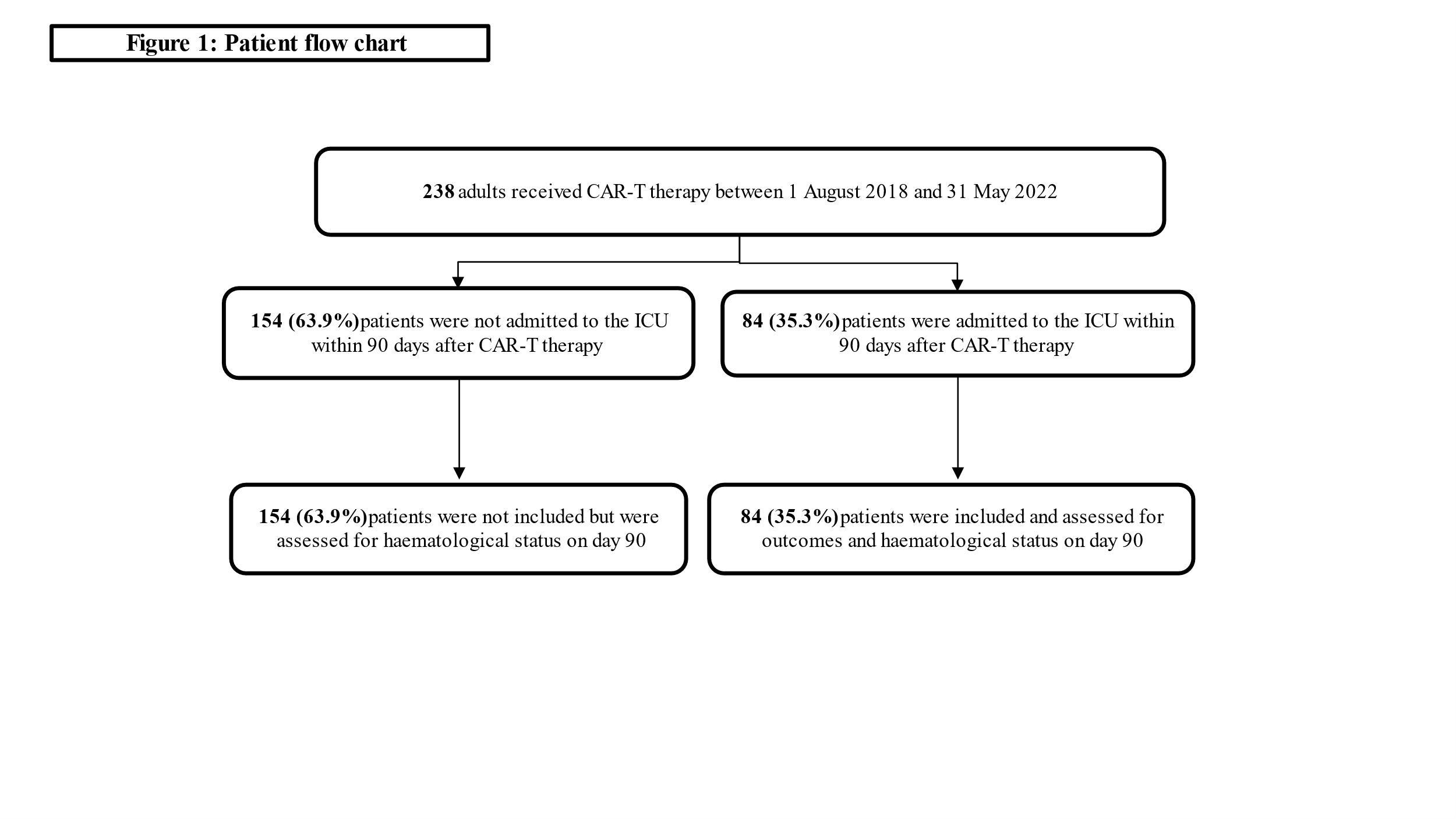
 CAR-T: Chimeric antigen receptor-T cells; ICU: intensive care unit

**Appendix 6: Infectious complications during the 97 ICU stays in 84 patients**

Table continues on the next page

| **Infectious complications** | **N (%)** |
| --- | --- |
| Microbiologically documented infection | 26 (26.8) |
| Clinically documented infection | 32 (33) |
| **Primary source of infection^a^** |  |
| Respiratory tract | 9 |
| Gastro-intestinal tract | 7 |
| Catheter | 6 |
| Bacteraemia | 5 |
| Urogenital tract | 4 |
| Cutaneous | 4 |
| Other^b^ | 4 |
| **Pathogen^c^** |  |
| *Escherichia coli* | 7 |
| *Pseudomonas aeruginosa* | 3 |
| *Enterococcus faecalis* | 3 |
| *Clostridioides difficile* | 3 |
| Other gram-positive cocci | 6 |
| Other gram-negative bacilli | 3 |
| Cytomegalovirus ^d^ | 2 |
| Fungi ^e^ | 3 |
| **Antimicrobial prophylaxis at ICU admission** | **N = 97 ICU stays** |
| Valaciclovir | 94 (96.9) |
| Trimethoprim-sulfamethoxazole | 83 (85.6) |
| Atovaquone | 11 (11.3) |
| Posaconazole | 19 (19.6) |
| Fluconazole | 21 (21.6) |
| **First-line antimicrobial treatment at ICU admission** | **N = 97 ICU stays** |
| Piperacillin-tazobactam | 25 (25.8) |
| Cefepime | 39 (40.2) |
| Carbapenems | 27 (27.8) |
| Vancomycin | 12 (12.4) |
| Linezolid | 9 (9.3) |
| Daptomycin | 6 (6.2) |
| Caspofungin | 8 (8.2) |
| Isavuconazonium sulphate | 4 (4.1) |
| Amphotericin B | 3 (3.1) |
| Aciclovir | 12 (12.4) |
| **Efficacy of empirical treatment** on the 26 microbiologically documented infections | 23 (88.5) |

^a^39 sources for 32 clinically documented episodes due to patients presenting with distinct concurrent infections

^b^Otorhinolaryngeal infection (n=3), encephalitis (n=1)

^c^30 pathogens for 26 microbiologically documented episodes due to patients presenting with distinct concurrent infections

^d^ Hemophagocytic lymphohistiocytosis with acute liver failure (n=1), encephalitis (n=1)

^e^Aspergillosis (n=1), *Mucorales* (n=2).

**Appendix 7: Additional data on patients with haemophagocytic lymphohistiocytosis (HLH) (n=15)**

Comparison of biological values between patients with or without HLH:

| *Median [IQR]* | **Patients with HLH (n=15)** | **Patients without HLH (n=69)** | *p* value |
| --- | --- | --- | --- |
| Ferritin (µmol/L) | 17189 (3891-41796) | 1577 (732-3562) | *<0.0001* |
| Triglycerids (g/L) | 3.8 (2.3-5.6) | 2.2 (1.3-2.7) | *0.0024* |
| Fibrinogen (g/L) | 0.85 (0.53-1.2) | 2.2 (1.4-3.8) | *<0.0001* |
| SGPT/SGOT (fold-increase) | 4 (1-5) | 1 (1-2) | *0.0068* |
| LDH (UI/L) | 484 (234-1513) | 220 (175-315) | *0.0018* |

Maximum values were reported (except for fibrinogen: minimum value); for SGPT/SGOT, the highest parameter was taken into account

Characteristics of patients with HLH (n=15):

|  | **N (%) or Median [IQR]** |
| --- | --- |
| **Haematological malignancy** |  |
| *Diffuse large B-cell lymphoma* | *5 (33.3)* |
| *B-cell acute lymphoblastic leukaemia* | *4 (27.7)* |
| *Multiple myeloma* | *4 (27.7)* |
| *Primary mediastinal large B-cell lymphoma* | *2 (13.3)* |
| **SOFA score** | **6 (4.25-9.75)** |
| **SAPSII score** | **41.5 (35-55)** |
| **Microbiologically documented infection** | **9 (60)** |
| **Life-sustaining therapy in the ICU** | **9 (60)** |
| *Vasopressors* | *9 (60)* |
| *Invasive mechanical ventilation* | *2 (13.3)* |
| *Renal replacement therapy* | *1 (6.7)* |
| **Specific treatment in the ICU** |  |
| *Corticosteroids* | *11 (73.3)* |
| *Tocilizumab* | *5 (33)* |
| *Second-line treatment* | *4 (27.7)* |
| **Outcomes** |  |
| *ICU mortality* | *2 (13.3)* |
| *Day-90 mortality* | *6 (40)* |
| *Day-90 progression / partial remission / complete remission* | *1 (6.7) / 0 (0) / 8 (53.3)* |

ICU: intensive care unit; SGOT: serum glutamo-acetoacetate transferase; SGPT: serum glutamic-pyruvite transferase; LDH: lactate deshydrogenase

Authors’ notes on these findings: all other data, including demographics, CAR-T product and previous medical history, etc., did not differ from the population without HLH. Among 9 patients with a documented infection: 4 had bacteriemia from a digestive source, 1 had *Clostridioides difficile* colitis, 2 had catheter-related bacteriemia, 1 had cytomegalovirus disease with acute liver failure, 1 had pulmonary aspergillosis. Second-line treatment was siltuximab (n=1), anakinra (n=1), siltuximab and anakinra (n=1), siltuximab and etoposide (n=1). As reported in Table 2 in the main manuscript, 7 HLH patients (46.7%) had disseminated intravascular coagulation, including one fatal case. The other patient who died in the ICU had multiorgan failure and concurrent septic shock. The 4 patients discharged alive who died before day 90 died of disease progression.

**Appendix 8: Use of second-line immunosuppressors**

Second-line treatment was given to 7/84 (8.3%) patients. All 7 patients had a worsening of their clinical condition despite a first-line of treatment, and all 7 of them were refractory to corticosteroid treatment.

- 1 patient had grade 4 ICANS with extensive myelitis and hemophagocytic lymphohistiocytosis and received anakinra;

- 3 patients had life-threatening grade 4 CRS with no microbiological documentation despite extensive explorations and received siltuximab (n=2) or anakinra (n=1);

- 3 patients had life-threatening hemophagocytic lympho-histiocytosis and received siltuximab (n=1), siltuximab and anakinra (n=1), siltuximab and etoposide (n=1).

**Appendix 9: Additional data on patients with repeated admissions**

Methods:

For patients admitted more than once during the study period, data were collected for each ICU stay. For all data reported per total number of patients, the occurrence of any categorical variable (death, infection, administration of a specific treatment or life-supporting intervention, etc.) was counted in the event of it happening in any stay. Concerning CRS and ICANS grading, the maximum severity reached on any stay was reported. Data reported per total number of stays are**:** reasons for ICU admission, SAPSII and SOFA scores, ICU length-of-stay, antibiotics usage and the details of infection findings (see e-Appendix 6).

Characteristics of patients with readmitted admissions (n=12):

|  | **N (%) or Median [IQR]** |
| --- | --- |
| **Haematological malignancy** |  |
| *Primary mediastinal large B-cell lymphoma* | *5 (41.7)* |
| *Diffuse large B-cell lymphoma* | *4 (33.3)* |
| *B-cell acute lymphoblastic leukaemia* | *3 (25)* |
| **Reason for ICU admission** |  |
| *Sepsis with microbiological documentation* | *6 (50)* |
| *ICANS relapse* | *4 (33.3)* |
| *Hemophagocytic lymphohistiocytosis* | *2 (16.7)* |
| **SOFA score** | **5 (4-7)** |
| **SAPSII score** | **47 (40-62)** |
| **Life-sustaining therapy in the ICU** | **7 (58.3)** |
| *Vasopressors* | *7 (58.3)* |
| *Invasive mechanical ventilation* | *2 (16.7)* |
| *Renal replacement therapy* | *2 (16.7)* |
| **Outcomes** |  |
| *ICU mortality* | *2 (16.7)* |
| *Day-90 mortality* | *2 (16.7)* |
| *Day-90 progression / partial remission / complete remission* | *0 (0) / 2 (16.7) / 8 (66.7)* |

ICU: intensive care unit

Authors’ notes on these findings: all other data, including demographics, CAR-T product and previous medical history, lengths of stay, etc., did not differ from the population with only one ICU stay. Sepsis was microbiologically documented from bacteriemia or digestive sources (Escherichia coli n=4, Pseudomonas aeruginosa n=2), and no patient had previous clinical or microbiological documentation of infection during their first stay. Among ICANS patients, 1 had grade 4 ICANS and 3 had grade 2 ICANS. Neither hemophagocytic lymphohistiocytosis patient had documented disease progression or infection during either stay. Exposure to tocilizumab or steroids during the first ICU stay did not differ from the population with only one stay. Tocilizumab was not used during subsequent ICU stays; steroids were used to treat ICANS and hemophagocytic lymphohistiocytosis patients, with favorable outcomes. No readmitted patient required life-sustaining therapy during their first stay. Invasive mechanical ventilation, renal replacement therapy and in-ICU death all occurred in two of the septic patients. One patient with grade 2 ICANS had a third ICU stay with favorable outcomes.

Another sensitivity analysis was performed, assessing features and outcomes of CAR-T patients based on data extracted from the first ICU stay only. The main findings are a lesser incidence of documented infection (17/84, i.e., 20.2%) and life-sustaining therapy (20/84, i.e., 23.8%; 16/84 had vasopressors, 10/84 had invasive mechanical ventilation, 2/84 had renal replacement therapy). There was no observable difference in tocilizumab (73/84, i.e., 86.9%) or corticosteroids usage (49/84 i.e., 58.3%). Mortality during the first ICU stay was 2.4% (2/84).

**Appendix 10: Causes of death in CAR-T recipients**

Regarding patients who died in the ICU (n=4): 2 died of septic shock during their second ICU stay; 1 died of septic shock due to ventilator-associated pneumonia after an extended ICU stay, in the context of grade 4 ICANS with super-refractory status epilepticus; 1 died of cerebral hemorrhage due to disseminated intravascular coagulation, secondary to severe haemophagocytic lymphohistiocytosis.

All patients discharged alive from the ICU who died before day 90 (n=10), as well as all patients who died before day 90 without ever being admitted to the ICU (n=16), died of disease progression.
